# Case report: preoperatively diagnosed perforated Meckel’s diverticulum containing gastric and pancreatic-type mucosa

**DOI:** 10.1186/s12893-017-0236-8

**Published:** 2017-04-11

**Authors:** Georges A. Abizeid, Hager Aref

**Affiliations:** International Medical Center, Jeddah, Saudi Arabia

**Keywords:** Meckel’s diverticulum, Perforated MD, Complicated MD

## Abstract

**Background:**

Meckel’s diverticulum is the most common congenital malformation of the gastrointestinal tract, and it represents a persistent remnant of the omphalomesenteric duct. Although it mostly remains silent, its infrequent occurrence is mirrored by the paucity of large series of data on it in the literature. Hemorrhage, obstruction and inflammation are most common complications of Meckel’s diverticulum. Perforation of Meckel’s diverticulum is considered very rare.

**Case presentation:**

We present the case of a 17-year -old male, who presented to the emergency department with 1-day history of lower abdominal pain. CT of the abdomen suggested a perforated Meckel’s diverticulum, which was confirmed later at the exploratory laparotomy. Perforation was due to progressive inflammation and presence of gastric and pancreatic tissue found on histopathology.

**Conclusion:**

Perforation of Meckel’s diverticulum is rarely suspected. Complications of Meckel’s diverticulum can be difficult to diagnose, and early recognition with timely operative intervention must occur in order to provide the best outcome for these patients. This is an interesting and unusual case of Meckel’s diverticulum perforation that highlights the importance of considering Meckel’s diverticulum as a differential diagnosis in every patient presenting with acute abdomen.

## Background

Meckel’s diverticulum (MD) has long been discussed in medical literature. MD was first mentioned by Fadricius Hildamus in 1598 [1]. It was named after the German anatomist Johann Friedrich Meckel, who described the embryological and pathological characteristics in an article published in 1809 [2]. MD is the most common congenital abnormality of the gastrointestinal tract, occurring in about 2% of the general population [3–5]. MD is a remnant of the omphalomesenteric duct, which is normally obliterated by the 5th to 8th week of gestation. It is a true diverticulum, containing all three layers of the bowel wall, and it arises from the antimesenteric border of the bowel. Only 2% of cases show symptoms, and is found twice as common in males than in females [6]. Most cases of MD are difficult to diagnose and are found incidentally during a surgical procedure for another reason. However, sometimes the presenting symptoms may guide the physician to suspect this pathology. The overall lifetime complication rate is approximately 4% [7]. The most common presentation is bleeding, followed by intestinal obstruction, diverticulitis, intussusception, neoplasm and perforation [8]. Perforation is very rarely seen, and it was reported in a review as being responsible for 0.5% of symptomatic MD [9]. MD perforation was reported to be a consequence of acute inflammation of MD [10]. Herein, we report a very rare case of perforated MD, which was identified prior to the operation. We provide an illustrative presentation, outlining one of the rare complications of MD in adults.

## Case presentation

**Fig. 1:**
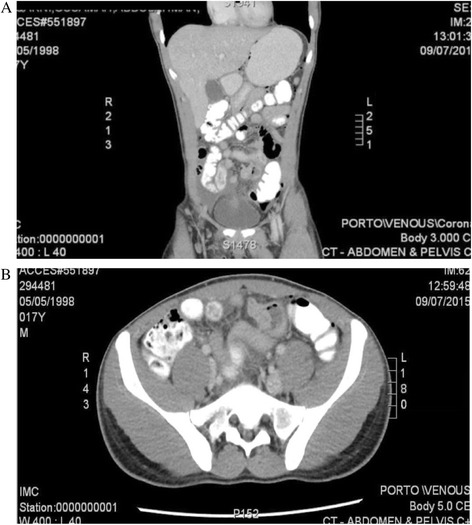
**a** and **b** views: CT scan of the abdomen and pelvis, showing an abnormal thickening of an ileal loop with possible presence of a MD. There are multiple air pockets suggesting an area of perforation at the thickened part of the ileal loop. (**a**: coronal view, and **b**: sagittal view)

A 17-year-old male patient, with no medical or surgical history, presented to the emergency department (ED) with 1-day history of lower abdominal pain that became more severe over the last 2 hours prior to his ED visit. The pain was colicky in nature, started around the umbilicus, and shifted to the right iliac fossa. It was associated with vomiting and nausea. There was no history of fever, diarrhea, or bleeding per rectum. **Clinical examination** revealed normal vital signs. The oral mucosa appeared dry, suggestive of dehydration. The abdomen was mildly distended. **On palpation**, the abdomen was soft and lax, with guarding and tenderness in the right iliac fossa and the umbilical areas. No masses were felt. Bowel sounds were exaggerated. **Laboratory** investigations were within normal range. Abdominal **X-rays** were unremarkable. **CT scan** of abdomen and pelvis demonstrated multiple pockets of intraperitoneal air, seen predominantly in the central and anterior abdomen, and signs of abnormal thickening of ileal loops with possible presence of a MD, (Fig. 1a and b). In addition, there was a normal appearing contrast opacified appendix and moderate ascites. The initial management included intravenous fluid resuscitation and antibiotic administration.

**Fig. 2:**
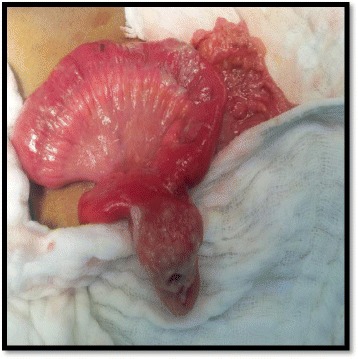
Intraoperative: Perforated MD with large base, located at 1 m from the ileocecal valve

**Fig. 3:**
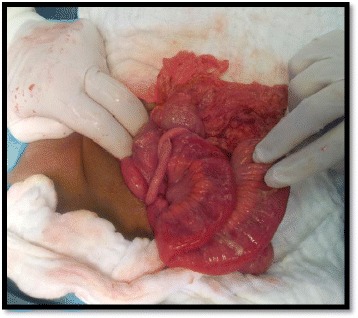
Intraoperative: Normal looking appendix

**Exploratory laparotomy** revealed a sessile and large base MD, inflamed and perforated at its tip, situated on the antimesenteric border at 1 m proximal to the ileocecal valve (Fig. 2). Appendix was normal (Fig. 3). Resection of the loop containing the MD with end-to-end anastomosis and appendectomy were performed.

**Fig. 4:**
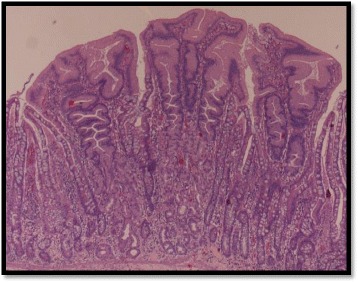
Histopathology: Heterotopic gastric tissue

**Fig. 5:**
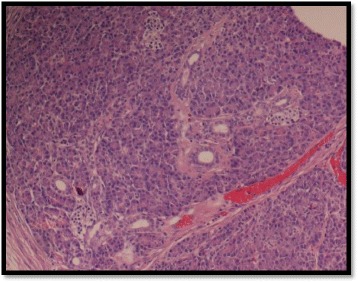
Histopathology: Heterotopic pancreatic tissue

**Histopathology** showed heterotopic gastric and pancreatic tissues with diverticulitis and no evidence of malignancy (Figs. 4 and 5). The patient had uneventful recovery.

The patient was followed up in the outpatient settings for 6 months following his operation. He has recovered and has no active complaints.

## Discussion

Meckel’s diverticulum is the most common congenital anomaly of the gastrointestinal tract [3–5, 11]. The **incidence** ranges between 1 and 2%, with a lifetime complication risk of 4–6% [8]. MD is a true diverticulum, usually found on the anti-mesenteric edge in the ileum [2, 12]. The majority of MD are asymptomatic and are incidentally discovered intraoperatively [13]. Perforation is reported to be a consequence of acute inflammation of MD, but the exact percentage of this pathology has not been reported. Ferguson et al. published in a search of Medline and Embase around 40 case-reports of perforated MD over the past 30 years [14]. Perforated MD may present as acute abdomen and resemble acute appendicitis [15]. It is either caused by irritation of foreign body, like fish bone [16, 17], bay leaf, chicken bone, needles and button battery [18–20]. or following blunt abdominal trauma, which was first described by Park and Lucas in 1970 [21]. Neoplastic causes, like GIST or leiomyoma, have been also reported [22, 23]. Perforation due to progressive inflammation of MD or ulcerating ectopic tissue was reported and was present in our case.

**Diagnosis** of MD is notably difficult, as the symptoms and imaging features are non-specific [9, 24]. CT scan and Ultrasound are not diagnostic because they can’t differentiate between a diverticulum and a loop of bowel [25]. Meckel-scan with 99mTc-pertechnetate may diagnose MD. It can detect the presence ectopic gastric mucosa in cases of complicated MD and can also identify the site of gastrointestinal bleeding. Its accuracy was reported to be around 90% in pediatric series, and only 46% in the adult group [26]. Less than 10% of symptomatic cases of MD are diagnosed preoperatively. In the reported case, perforated MD was diagnosed preoperatively on CT scan, which makes our case exceptional [27].

Surgical resection is considered the **treatment** of choice for the symptomatic MD. This can be achieved by diverticulectomy, segmental bowel resection and anastomosis and wedge resection. This is especially applicable when there is palpable ectopic tissue at the diverticular-intestinal junction, intestinal ischemia or perforation. In the reported case, the patient had perforation of a sessile MD. Resection of the involved bowel segment and anastomosis was indicated.

## Conclusion

Meckel’s diverticulum perforation is very rare; however, it should be kept in mind as a differential diagnosis for every patient presenting with acute abdomen. In cases where the nature of the complication is likely to require surgical management, an early laparoscopic or open exploration should be performed in order to prevent the morbidity and mortality associated with late complications. The treatment should be based on the surgeon’s judgment and on the inherent characteristics of each patient.

## Abbreviations

CT: Computed tomography
ED: Emergency department
GIST: Gastrointestinal stromal tumor
IMC: International medical center
MD: Meckel’s diverticulum
